# The Diagnostic Value of the Correlation between Serum Anti-p53 Antibody and Positron Emission Tomography Parameters in Lung Cancer

**DOI:** 10.4274/mirt.97269

**Published:** 2016-09-29

**Authors:** Zekiye Hasbek, Ömer Tamer Doğan, İsmail Sarı, Birsen Yücel, Mehmet Metin Şeker, Bülent Turgut, Serdar Berk, Yavuz Siliğ

**Affiliations:** 1 Cumhuriyet University Faculty of Medicine, Department of Nuclear Medicine, Sivas, Turkey; 2 Cumhuriyet University Faculty of Medicine, Department of Chest Diseases, Sivas, Turkey; 3 Cumhuriyet University Faculty of Medicine, Department of Biochemistry, Sivas, Turkey; 4 Cumhuriyet University Faculty of Medicine, Department of Radiation Oncology, Sivas, Turkey; 5 Turgut Özal University Faculty of Medicine, Department of Medical Oncology, Ankara, Turkey

**Keywords:** Anti-p53 antibody, Lung Cancer, fluorodeoxyglucose, positron emission tomography/computed tomography

## Abstract

**Objective:**

Mutations in the p53 gene are the most commonly observed genetic abnormalities in malignancies. The purpose of this study was to assess the diagnostic value of serum anti-p53 antibody (Ab) along with the correlation between serum anti-p53 Ab level and quantitative positron emission tomography (PET) parameters such as maximum standardized uptake value (SUV_max_), SUV_av_e, metabolic tumor volume, total lesion glycolysis (TLG) and tumor size.

**Methods:**

Serum anti-p53 Ab level was studied in three groups. Patients who underwent ^18^F-fluorodeoxyglucose (FDG) PET/computed tomography (CT) imaging for staging of previously diagnosed lung cancer constituted the first group, while patients who underwent ^18^F-FDG PET/CT imaging for evaluation of suspicious pulmonary nodules detected on thorax CT and did not show pathologic FDG accumulation (NAPN=pulmonary nodule with non avid-FDG) were enrolled in the second group. The third group consisted of healthy volunteers.

**Results:**

Twenty-eight patients with lung cancer (median age: 62.5, range: 39-77years), 28 patients with NAPN (median age: 65, range: 33-79 years), and 24 healthy volunteers (median age: 62, range: 44-74 years) were enrolled in the study. The serum anti-p53 Ab level was low in healthy volunteers while it was higher in both lung cancer patients and NAPN patients (p<0.05). When serum anti-p53 Ab level and PET parameters were evaluated, there was no significant correlation between serum anti-p53 Ab level and SUV_max_, SUV_ave_, TLG, tumor volume and tumor size of patients with lung cancer (p>0.05). Besides, there was no significant difference between serum anti-p53 Ab level and lesion size of NAPN patients (p>0.05).

**Conclusion:**

It was determined that serum anti-p53 Ab levels are not significantly correlated with PET parameters, and that serum anti-p53 Ab levels increase in any benign or malignant lung parenchyma pathology as compared to healthy volunteers. These results indicate that this Ab cannot be used as a predictor of malignancy in a lung lesion.

## Abstract (tr)

**Amaç:**

p53 gen mutasyonları malignitelerde en sık görülen genetik bozukluktur. Bu çalışmada serum anti-p53 antikor (Ab) düzeyinin tanısal değerinin araştırılması, serum anti-p53 Ab düzeyi ile SUV_max_, SUV_ave_, metabolik tümör volümü, total lezyon glikolizi (TLG) ve tümör boyutu gibi kantitatif pozitron emisyon tomografisi (PET) parametreleri arasındaki ilişkinin değerlendirilmesi amaçlanmıştır.

**Yöntem:**

Serum anti-p53 Ab düzeyi üç grupta çalışıldı. Birinci grupta akciğer kanseri nedeniyle evreleme için ^18^F-florodeoksiglukoz (FDG) PET/bilgisayarlı tomografi (BT) görüntülemesi yapılan hastalar, ikinci grupta ise toraks BT’de şüpheli pulmoner nodül saptanması nedeniyle tanı amacıyla ^18^F-FDG PET/BT yapılan ancak patolojik FDG tutulumu izlenmeyen hastalar (NAPN=FDG tutmayan pulmoner nodül) vardı. Üçüncü grubu sağlıklı gönüllüler oluşturdu.

**Bulgular:**

Bu çalışmaya akciğer kanseri olan 28 hasta (ortalama yaş: 62,5, aralık: 39-77 yıl), NAPN olan 28 hasta (ortalama yaş: 65, aralık: 33-79 yıl), ve 24 sağlıklı gönüllü (ortalama yaş: 62, aralık: 44-74 yıl) dahil edildi. Serum anti-p53 Ab düzeyi sağlıklı gönüllülerde düşük iken, akciğer kanserli hastalarda ve NAPN hastalarında yüksekti (p<0,05). Serum anti-p53 Ab düzeyi ve PET parametreleri değerlendirildiğinde; serum anti-p53 Ab düzeyi ile SUV_max_, SUV_ave_, TLG, tümör volümü ve akciğer kanserli hastalardaki tümör boyutu arasında anlamlı korelasyon saptanmadı (p>0,05). Ayrıca serum anti-p53 Ab düzeyi ile NAPN hastalarındaki lezyon boyutu arasında anlamlı farklılık yoktu (p>0,05).

**Sonuç:**

Serum anti-p53 Ab düzeyi ile PET parametreleri arasında anlamlı korelasyon olmadığı ve serum anti-p53 Ab düzeyinin akciğerde benign veya malign herhangi bir parankim patolojisinde yükseldiği bulundu. Bu sonuçlar serum anti-p53 Ab’nin malign akciğer lezyonları için bir belirteç olamayacağını düşündürmektedir.

## INTRODUCTION

Lung cancer comprises an important group of malignancies, and is the most common cause of cancer-related deaths with a short survival after initial diagnosis. About 90% of primary lung cancers are non-small cell cancer (NSCLC) (1). Accurate staging is important for determining the choice of treatment and predicting prognosis. ^18^F-fluorodeoxyglucose (FDG) positron emission tomography/computed tomography (PET/CT) has an important role in staging, and it is the most advanced imaging technique that has been developed to determine the metabolic characterization of the tumor. Maximum standardized uptake value (SUV_m_ax), which is acquired by PET imaging, is commonly used in clinical practice as a criterion for malignancy. A high SUV_max_ value is commonly accepted as a poor prognostic factor (2). Due to the development of new software programs, recent studies have shown that metabolic tumor volume (MTV) and total lesion glycolysis (TLG) may be useful quantitative parameters for prognostic evaluation (3). Besides, some reports have suggested that TLG is a better prognostic indicator than SUV_max_ (4).

Recently, there has been an increase in studies on molecular biology of cancer. Prognostic tests have been developed especially for lung cancer, and it has been reported that the presence of auto-antibodies may be used for disease diagnosis at an early stage (5). p53 is a tumor suppressor gene that plays an important role in controlling normal cell proliferation, and is located on the chromosome 17 (17p13.1). It is the most common target of genetic alteration in many tumors (6). Serum p53 protein is present in normal healthy individuals. However, serum anti-p53 antibody (Ab) is extremely rare (7). Mutations in p53 gene cause an accumulation of non-functional proteins and development of anti-p53 Abs, which can be detectable in tissues, blood, slouched cells and other body fluids (8). In many studies conducted up to present, it has been shown that p53 mutation was more frequent in lung, esophagus, stomach, bone, bladder, ovarian, and brain (except glioma) cancers as well as lymphoma and leukemia (8). The rate of p53 gene mutation is higher in patients with small cell lung cancer (SCLC) as compared to NSCLC patients (9). In the literature, there are some publications on the role of p53 gene mutation in the early diagnosis of lung cancer (10).

The purpose of this study was to assess the diagnostic value of serum anti-p53 Ab along with the correlation between serum anti-p53 Ab level and quantitative PET parameters such as SUV_max_, SUV_ave_, MTV, TLG and tumor size.

## MATERIALS AND METHODS

### Patients

Patients who were referred to our department with the purpose of ^18^F-FDG PET/CT imaging for staging of previously diagnosed lung cancer and patients who underwent ^18^F-FDG PET/CT imaging for evaluation of suspicious pulmonary nodules detected on thorax CT and did not show pathologic FDG accumulation (NAPN=pulmonary nodule with non avid-FDG) were enrolled in this prospective and preliminary study. The control group consisted of healthy subjects with no known history of cancer and without any complaints. Only serum anti- p53 Ab was measured in the control group. This study was performed in accordance with the principles of the Declaration of Helsinki. This study was approved by the local ethical committee (2013-03/50). Oral and written consent was obtained from all patients. Patients with previous operations for lung cancer, prior chemotherapy or radiotherapy for lung cancer, those with no definitive histological diagnosis, and those with a blood glucose level greater than 150 mg/dL were excluded from the study. Clinicopathological data of the patients are reported in Table 1.

### Detection of Serum Anti-p53 Antibodies

Blood samples were collected from peripheral blood vessels. Blood samples were taken before performing the FDG administration for ^18^F-FDG PET/CT. All serum was obtained after complete clotting by centrifugation, immediately frozen and stored at -80 °C until the time of analysis. Serum anti-p53 Abs levels were measured using Enzyme-linked immune-sorbent assay kit (CUSABIO) according to the manufacturer’s instructions. The kit was designed to measure circulating p53 antibodies in human serum samples.

### Imaging Acquisitions/Analysis

PET imaging was performed prior to any treatment and images were acquired using a combined PET/CT scanner (Discovery 600 PET/CT GE Medical Systems, USA). Each patient fasted for at least 6 h before imaging. After ensuring that blood glucose was <150 mg/dl, approximately 370 MBq ^18^F-FDG were administered intravenous 1 hour before image acquisition and patients were rested. Attenuation correction of PET images with CT data was performed. First, the CT scan was performed. CT images were acquired with 70 mA, 120 kV, axial slice thickness of 3.75 mm. Right after CT image acquisition, a standard PET imaging protocol was applied from the cranium to the mid-thigh with an acquisition time of 3 min/bed in 3-dimensional mode. All PET studies were acquired in 3-D mode. CT and PET images were matched and fused into transaxial, coronal and sagittal images. The data were transferred via the Digital Imaging and Communications in Medicine protocol to a processing Workstation (AW Volumeshare 5 GE Medical Systems S.C.S, France), and visual and semi-quantitative analyses were performed. Tumor size (three maximum diameters) was measured. SUV_max_, SUV_mean_ and MTV were calculated from attenuation-corrected ^18^F-FDG PET images for tumor mass. The SUV_max_ was calculated by using the following formula: maximum pixel value with the return on investment activity (MBq/kg)/ [injected dose (MBq)/body weight (kg)]. SUV_m_ean was determined from the average voxel counts within the tumor region. Automatic volume of interest using an isocontour threshold method was placed over the primary tumor. TLG was then calculated as: “TLG=SUV_mean_ x MTV”.

### Statistical Analysis

The statistical tests were performed using SPSS (version 14.0; SPSS, Inc.). The frequencies test was applied to evaluate the statistical significance of the parameters. Correlations among serum anti-p53 Ab levels, tumor volume, tumor size, SUV_mean_, SUV_max_, and TLG were examined by the Pearson correlation test. Correlation between serum anti-p53 Ab levels and case groups, histopathologic subtype of cancer patients and stage of cancer were evaluated by using Kolmogorov-Smirnov test since parametric hypothesis could not be tested. Correlation between serum anti-p53 levels and tobacco smoking status, age and sex of cases were evaluated with Mann-Whitney U test. Significance levels were presented as p values. It was assumed that the observed differences were statistically significant at the p<0.05 levels.

## RESULTS

Twenty-eight lung cancer patients (median age: 62.5, range: 39-77 years), 28 patients with NAPN (median age: 65, range: 33-79 years), and 24 healthy volunteers (median age: 62, range: 44-74 years) were included in this preliminary, prospective study.

According to histopathology results; 18 (64.3%) squamous cell carcinomas, nine (32.1%) adenocarcinomas and one (3.6%) large-cell carcinoma patients were included. The median primary tumor size in patients with lung cancer (n=28) was 52.9 mm (range: 19.6-92.4 mm). The lesion size in NAPN patients (n=28) was 16 mm (range: 10-51 mm). The subjects consisted of 26 (92.9%) males and two (7.1%) females, 21 (75%) males and seven (25%) females, 21 (87.5%) males and three (12.5%) females in lung cancer, NAPN, and healthy volunteers, respectively.

**Figure 1:**
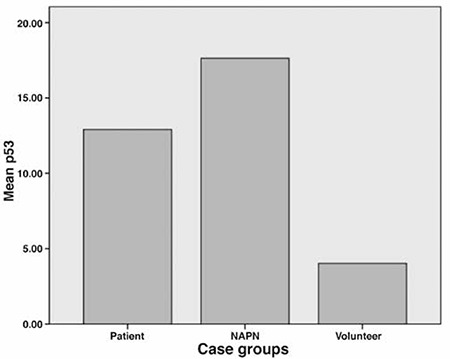
Comparison of serum anti-p53 antibody levels of cases included in the study NAPN: Pulmonary nodule with non avid-fluorodeoxyglucose

The median serum anti-p53 Ab value was 3.75 ng/mL (range 2.23-104.19 ng/mL) in all cases. The median serum anti-p53 Ab level in epidermoid cancer group, adeno-carcinoma group, and large-cell carcinoma group was 4.10 ng/mL (range: 2.67-52.49 ng/mL), 4.16 ng/mL (3.26-104.19 ng/mL) and 4.5 ng/mL, respectively. The median serum anti-p53 Ab level in lung cancer group, NAPN group and healthy volunteer group was 12.90 ng/mL (range: 2.67-104.19 ng/mL), 17.63 ng/mL (2.52-92.19 ng/mL) and 4.02 ng/mL (2.23-25.72 ng/mL), respectively. Serum anti-p53 levels were higher in lung cancer and NAPN patient groups in comparison with healthy volunteers (p=0.0001). Figure 1 shows the comparison of serum anti-p53 Ab levels of all cases included in the study. Clinical details of all cases and their relation with serum anti-p53 Ab levels were summarized in Table 2.

Serum anti-p53 Ab level was measured as 25 ng/mL in one healthy volunteer while the mean serum anti-p53 Ab level was 3.08±0.43 ng/mL (range: 2.23-4.17 ng/mL) in other healthy volunteers. This volunteer had a history of smoking for almost 45 years. A thorax CT was performed to that volunteer that did not show any mass or nodule. However, emphysema was detected in lung parenchyma.

Evaluation of serum anti-p53 Ab level and PET parameters showed that there was no significant correlation between serum anti-p53 Ab level and SUV_max_, SUV_ave_, TLG, tumor volume, and tumor size of patients with lung cancer (p=0.189, 0.123, 0.572, 0.928, 0.421, respectively). Besides, there was no significant difference between serum anti-p53 Ab level and lesion size of NAPN patients (p=0.694). There was no association between serum anti-p53 Ab level and age or sex (p=0.704, p=0.771, respectively). Moreover, there was no significant correlation between histopathologic subtypes or stage of cancer and serum p53-Ab levels (p=0.920, p=0.847, respectively). In the lung cancer group, the mean serum anti-p53 Ab level was higher in stage I patients at the time of diagnosis in comparison with other stage groups; however, the difference was not statistically significant (p=505). On the other hand, there was significant correlation between serum anti-p53 Ab levels and tobacco smoking (p=0.011) (Table 2).

Two patients included in the study had a previous history of cancer. Both patients were in complete remission and follow-up. One of them had colon cancer and the other had renal cell carcinoma. While serum anti-p53 Ab level was 52.49 ng/mL in the patient with colon cancer, it was 3.45 ng/mL in the patient with renal cell carcinoma.

## DISCUSSION

Cancer is among the most serious health problems all over the world. While scientists continue their studies on development of novel cancer treatments, studies on the detection of cancer before it manifests or at an early stage are also being performed. With this purpose, by developing panels of tumor-associated antigens, it is being investigated if these auto-antibodies can be used as markers (11). Lung cancer, as being the most common cause of cancer-related death, is one of the most researched cancer type in this regard.

A p53 mutation appears to be the most common genetic defect identified in malignancies (12). Antibodies against p53 can be detected in the serum. Some studies suggested that serum p53-Ab can be used as a tumor marker (8). In this study, we investigated the correlation between serum anti- p53 Ab and PET parameters in lung cancer patients. In our study, mean serum anti-p53 Ab level was higher in patients with lung cancer and those with non-avid FDG pulmonary nodules in comparison to healthy volunteers. However, there was no significant difference between serum anti-p53 Ab levels of patients with lung cancer and patients with NAPN. These results indicate that this Ab is not specific for lung cancer. Besides, these findings raise the suspicion that any situation inducing hypoxia in the lung (such as lung cancer, solitary pulmonary nodule, or obstructive lung diseases like emphysema) might lead to an increase in serum anti-p53 Ab levels. The number of studies evaluating the correlation between p53 mutation and prognosis in lung cancer has been increasing, but the results of these studies are equivocal. Likewise, there are many studies in the literature on the role of serum anti-p53 Abs in the early diagnosis of lung cancer as well as other cancer types. While some studies practice on panels of other antigens in addition to p53, some studies practice the sensitivity of anti-p53 Abs assay methods (13). Maddau et al. (12) detected p53 expression at 51.7% of 180 NSCLC patients. They reported that patients with p53 and Ki-67 overexpression had worse outcome in stage I adenocarcinoma (but not those with stage II and III or other histologic types). They stated that the effects of p53 alteration may depend on tumor cell type. In our study, the mean serum anti-p53 Ab level in stage I patients was found to be higher than that in other stages (Table 2), although not statistically significant. Ciancio et al. (6) studied anti-p53 and anti-Ki-67 antibodies within specimens obtained by fiber-optic bronchial biopsies, and they reported that the prognosis was better in Ab-negative patients. Gomez et al. (14) reported that p53 protein overexpression in lung adenocarcinoma correlated with lymphatic vessel invasion. Gao et al. (15) also detected p53 mutation in 20% of adenocarcinoma cases and 35% of squamous cell carcinoma cases in their study. In addition to that, although they could not find any correlation between p53 mutation and survival, they found that tumor size and degree of differentiation were predictors of poor survival. Chapman et al. (5) reported that the presence of tumor-associated autoantibodies may be useful in the early prognosis of lung cancer and that it may be used especially on high risk individuals. Similarly, in another study, p53 suprabasal immunostaining was detected in 75% of severely dysplastic or carcinoma in situ lesions, and a “contribution value to predict the biological behavior of pre-neoplastic endobranchial lesions” was expressed (16). On the other hand, it has been reported that the presence of p53 Ab is not cancer specific and that it was also found to be positive in patients with impaired lung function. (17). Moreover, the prognostic role of anti-p53 antibodies in lung cancer remains controversial, as some studies indicate an association with poor prognosis others report no correlation between survival and anti-p53 Abs (18).

SCLC progresses more aggressively than NSCLC since it has a tendency to present with disseminated metastasis at the time of diagnosis. It is also the most responsive type of tumor to treatment as compared to other lung cancer patients. Chapman et al. (11) evaluated SCLC patients for the presence of autoantibodies against certain antigens (p53, CAGE, NY-ESO-1, GBU4-5, Annexin I, SOX2, and Hud), and they found that antibodies existed at least to one of the 6 antigens. The specificity was found to be 90% when compared with the control group, while the specificity was reported to be greater than 99% in the limited panel composed of p53, SOX2 and Hu-D. Park et al. (19) also reported that together with anti-p53 other conventional markers help to increase sensitivity and specificity in lung cancer detection.

Tobacco smoking is still one of the major risk factors inducing lung cancer. Gibbons et al. (20) reported that although p53 mutation rate is high in smoking-related tumors such as lung, bladder, oral cavity and conductive airways, tobacco smoking also produces mutation of the tumor suppressor TP53. In our study, when cases were considered altogether, there was a significant correlation between serum anti-p53 Ab levels and smoking. However; when lung cancer, NAPN, and volunteer groups were considered separately, there was no significant difference between serum anti-p53 Ab levels. Hypoxia induces p53 protein accumulation and p53 dependent apoptosis in oncogenically transformed cells (21). Hypoxic cells might be the reason of elevation in serum anti-p53 Ab levels in smokers. In one healthy volunteer in our study, the serum anti-p53 Ab level was higher in comparison to the mean serum anti-p53 Ab levels of other healthy volunteers. This volunteer had a smoking history of almost 45 years. The thorax CT performed on that volunteer who had no complaint of coughing, sputum, labored breathing or exertion dyspnea revealed emphysema.

Conventionally, CT has been the primary imaging technique for the diagnosis and staging of lung cancer patients. Similar to normal cells, tumor cells also use glucose for energy production. Therefore, recent advances in combined PET/CT imaging systems have further improved staging and disease management based on the metabolic information in addition to anatomic imaging. Routinely, the most commonly used measure of FDG uptake is the SUV. For this reason, SUV is usually used as a tumor marker indicating tumor aggressiveness, especially in adenocarcinoma (22). SUV is a simple, practically applicable and repeatable parameter. On the other hand, it is time-dependent and affected by several factors such as glucose metabolic rate and partial volume effect (23). That is why the importance of PET parameters such as TLG and MTV is being emphasized for prognostic evaluation (24). In the literature, there are some publications suggesting that the mutation of tumor suppressor genes such as p53 is correlated with an increase in FDG uptake in lung cancer (25). Duan et al. (26) reported that p53 expression is the primary predictive factor for SUV_max_. Zhou et al. (27) found a significant difference between SUV_max_ and Tp53-induced glycolysis and apoptosis regulator (TIGAR)’s being positive or negative. On the other hand, Watanabe et al. (28) did not find a correlation between p53 alteration and FDG uptake. Similarly in our study, there was no significant correlation between serum anti-p53 Ab level and PET parameters such as SUV_m_ax, SUV_ave_, TLG, and tumor volume. However, this situation might occur because of FDG uptake in tumor tissue is dependent on hypoxia in tumor cell. Hypoxic cancer cells were reported to have significantly higher radiolabeled FDG uptake than normoxic cancer cells in animal studies (29). Unfortunately, the hypoxia condition of tumor cells was not evaluated in our study. Another limitation of the study comprises the relatively small number of patients.

## CONCLUSION

Based on the results, we have found that serum anti-p53 Ab level does not correlate with PET parameters, and that serum anti-p53 Ab level increases in any benign or malignant lung parenchyma pathology as compared to healthy volunteers. These results suggest that this Ab cannot be used as an indicator for malignant lung lesions. 

Principally, tobacco smokers and people who work in jobs that highly expose them to carcinogens are well aware of their risk to get cancer. As researchers, are we doing the right thing by introducing additional tests like serum anti-p53 Ab level that cause healthy people more distress on the probability of having lung cancer at any time? Would it be more appropriate to use these tests only on patients with suspected malignancy for early diagnosis until a test with very high sensitivity and specificity is identified? Currently, detecting cancer before it manifests clinically is impossible. However, we believe that future studies on tumor markers like anti-p53 Ab will reveal important tools as reagents of early detection of metastasis/recurrence in previously/already diagnosed cancer patients.

## Ethics

Ethics Committee Approval: The study was approved by the Cumhuriyet University of Local Ethics Committee (2013-03/50), Informed Consent: Consent form was filled out by all participants.

Peer-review: Externally peer-reviewed.

## Figures and Tables

**Table 1:**
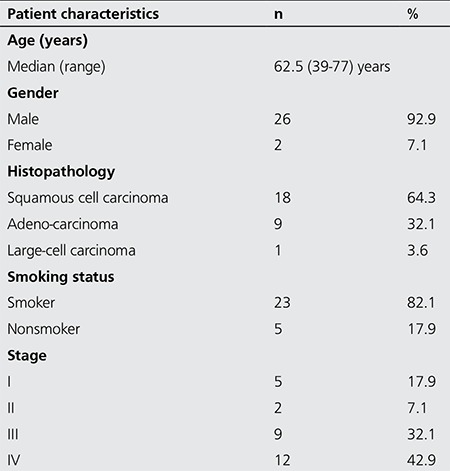
Lung cancer patient’s characteristics

**Table 2:**
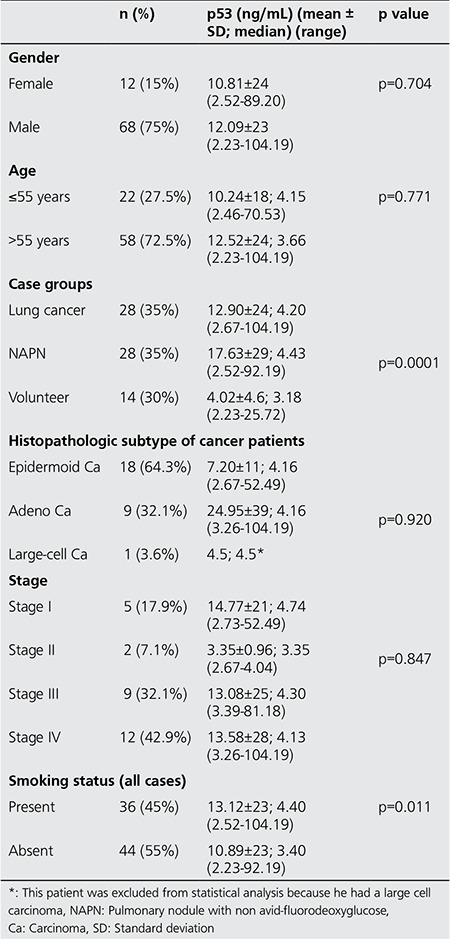
Characteristics of the entire study group and comparison of serum anti-p53 antibody levels within sub-groups
